# Glycosylated clusterin species facilitate Aβ toxicity in human neurons

**DOI:** 10.1038/s41598-022-23167-z

**Published:** 2022-11-03

**Authors:** Evangeline M. Foster, Marco Fernandes, Adria Dangla-Valls, Philip Hublitz, Menelaos Pangalos, Simon Lovestone, Elena M. Ribe, Noel J. Buckley

**Affiliations:** 1 Department of Psychiatry, Warneford Hospital, University of Oxford, Oxford, OX3 7JX UK; 2 MRC Weatherall Institute of Molecular Medicine, Genome Engineering Facility, University of Oxford, John Radcliffe Hospital, Headley Way, Oxford, OX3 9DS UK; 3 Evotec International GmbH, Marie-Curie-Straße 7, 37079 Göttingen, Germany; 4 BioPharmaceuticals R&D, AstraZeneca, Melbourn Science Park, Cambridge Road, Royston, SG8 6EE Herts UK; 5 Kavli Institute for Nanoscience Discovery, University of Oxford, Oxford S Parks Rd, Oxford, OX1 3QU UK; 6 Present Address: Janssen Medical, High Wycombe, UK 50-100 Holmers Farm Way, HP12 4EG

**Keywords:** Stem cells, Neuroscience, Alzheimer's disease

## Abstract

Clusterin (*CLU*) is one of the most significant genetic risk factors for late onset Alzheimer’s disease (AD). However, the mechanisms by which CLU contributes to AD development and pathogenesis remain unclear. Studies have demonstrated that the trafficking and localisation of glycosylated CLU proteins is altered by *CLU*-AD mutations and amyloid-β (Aβ), which may contribute to AD pathogenesis. However, the roles of non-glycosylated and glycosylated CLU proteins in mediating Aβ toxicity have not been studied in human neurons. iPSCs with altered CLU trafficking were generated following the removal of *CLU* exon 2 by CRISPR/Cas9 gene editing. Neurons were generated from control (CTR) and exon 2 −/− edited iPSCs and were incubated with aggregated Aβ peptides. Aβ induced changes in cell death and neurite length were quantified to determine if altered CLU protein trafficking influenced neuronal sensitivity to Aβ. Finally, RNA-Seq analysis was performed to identify key transcriptomic differences between CLU exon 2  −/− and CTR neurons. The removal of *CLU* exon 2, and the endoplasmic reticulum (ER)-signal peptide located within, abolished the presence of glycosylated CLU and increased the abundance of intracellular, non-glycosylated CLU. While non-glycosylated CLU levels were unaltered by Aβ_25–35_ treatment, the trafficking of glycosylated CLU was altered in control but not exon 2  −/− neurons. The latter also displayed partial protection against Aβ-induced cell death and neurite retraction. Transcriptome analysis identified downregulation of multiple extracellular matrix (ECM) related genes in exon 2  −/− neurons, potentially contributing to their reduced sensitivity to Aβ toxicity. This study identifies a crucial role of glycosylated CLU in facilitating Aβ toxicity in human neurons. The loss of these proteins reduced both, cell death and neurite damage, two key consequences of Aβ toxicity identified in the AD brain. Strikingly, transcriptomic differences between exon 2  −/− and control neurons were small, but a significant and consistent downregulation of ECM genes and pathways was identified in exon 2  −/− neurons. This may contribute to the reduced sensitivity of these neurons to Aβ, providing new mechanistic insights into Aβ pathologies and therapeutic targets for AD.

## Introduction

Alzheimer’s disease (AD) is a progressive neurodegenerative disorder and is the most common cause of dementia^1^. AD is characterised by the presence of two pathological hallmarks: extracellular Aβ aggregates known as Aβ plaques and intracellular neurofibrillary tau tangles (NFTs) formed of hyperphosphorylated tau proteins. A prevailing hypothesis of AD is the Amyloid Cascade Hypothesis that postulates Aβ plays a central role in initiating AD development^2–4^. Aggregation of Aβ may trigger downstream events, including synaptic dysfunction, neurite damage, formation of NFTs and cognitive decline that ultimately result in AD. Major support for this hypothesis arises from the genetics of familial AD (fAD)^2^ where several mutations in genes encoding proteins involved in Aβ production, including amyloid precursor protein (*APP*), presenilin 1 and 2 (*PSEN 1/2*)^5–12^ have been identified. However, fAD represents only a small number of AD cases (< 1%), and it is not clear if fAD offers mechanistic insight into sporadic AD (sAD). Nevertheless, such studies provide important insight into a crucial role of Aβ in AD development. Numerous risk genes have been identified that alter an individual’s susceptibility to sAD, of which *CLU* is the third most significant genetic risk factor after *APOE* and *BIN1*^13–15^*.* However, little is known of the mechanisms by which single nucleotide polymorphisms (SNPs) and mutations in CLU alter AD risk^13,14,16^. Although rare *CLU* mutations have been identified to alter CLU protein localisation, these are non-coding and located in exons 5 and 6^16–18^. To date no AD-related *CLU* variant has been described in exon 2^19^.

In addition to *CLU* acting as a genetic risk factor for sAD, much interest has been placed on CLU’s relationship with Aβ. Secreted CLU binds to Aβ altering its solubility and aggregation^13–16^ and contributes to the clearance of Aβ by astrocytes and through the blood–brain barrier^21^. In contrast, data from animal studies have identified a role for CLU in facilitating the deposition of Aβ and the formation of plaques^20,22,23^, suggesting CLU may facilitate AD pathology in vivo. It therefore remains unclear if CLU proteins facilitate or protect against Aβ toxicity in AD.

Clusterin is a highly glycosylated, cleaved and secreted protein (sCLU) that is considered to be cytoprotective^21–23^. The biogenesis of sCLU is well described^24–28^. Initially, *CLU* mRNA is translated into a clusterin preproprotein that is trafficked to the ER and the Golgi apparatus, where it is cleaved, phosphorylated and glycosylated^29^. Located within exon 2 is the endoplasmic reticulum (ER) signal peptide that directs the trafficking of immature CLU protein to the ER and subsequently to the Golgi apparatus where it is cleaved, phosphorylated and glycosylated to generate mature and cleaved, secreted CLU proteins^28^. Additionally, inside the ER, the ER signal peptide is removed to generate an N-terminal truncated protein. Mature clusterin (75/80 kDa) is secreted as a heterodimeric protein consisting of an α chain and a β chain that are linked by 5 disulphide bonds^24,27,28,30–32^. Although typically considered secreted, glycosylated CLU proteins have also been identified within cells, typically in the cytoplasm, although the function, origin and biogenesis of intracellular, glycosylated CLU proteins remains debated^33^.

Stress has been demonstrated to induce localisation and trafficking of glycosylated CLU into the cytoplasm. This may be mediated through the binding of sCLU to cell surface receptors resulting in its uptake back into cells^34,35^, or via premature release of CLU from the secretory pathway prior to secretion^36^, both of which result in reduced secretion and increased intracellular retention of CLU proteins^35–40^. Altered CLU trafficking in vitro has been demonstrated in response to a variety of stressors, including ER stress^36,38,40^, proteasomal inhibition^41^, nerve growth factor^39^. With relevance specifically to AD, the treatment of cells with Aβ^35,37^ and *CLU*-AD mutations^17,18^ have both been demonstrated to alter CLU trafficking suggesting CLU trafficking contributes to AD pathogenesis. Clusterin trafficking is altered by Aβ_25–35_ treatment of rodent neurons^35^ and by Aβ_1–42_ treatment of astrocytes in vitro^37^, both of which lead to increased intracellular abundance and reduced CLU secretion. The observed alteration of CLU trafficking in rodent neurons suggested CLU trafficking may mediate Aβ induced toxicity in these cells, which was further supported by the demonstration that *CLU* knockdown protects rodent neurons against Aβ induced cell death^35^. Furthermore, several *CLU* mutations enriched in AD cohorts^16^, were demonstrated to alter the trafficking and subcellular localisation of clusterin demonstrating a potential mechanism by which *CLU* variants may increase the risk of developing AD^17,18^. Thus, several types of stressors in different cell types have been shown to alter CLU trafficking, however, it remains unclear if this alteration in trafficking facilitates cellular stress or a protective response in human neurons.

Although often referred to as a cytoprotective protein, CLU has also been shown to play a pro-apoptotic role in the human breast cancer cell line MCF-7^42,43^. Alternative splicing of *CLU* generates a minor *CLU* variant lacking exon 2 (also referred to as *CLU* variant 1 Δ exon 2)^41–43^. Translation of this variant generates a non-glycosylated, uncleaved CLU protein that resides intracellularly, typically in the cytoplasm and nuclei of cells^41–43^. Although this shorter CLU isoform has low abundance in cells^41,43^ or is not expressed at all^44^, expression is upregulated in cancer cell lines by stress and following induction of the cell death cascade, suggesting a role for these proteins in mediating cellular response to stress^41,43^. However, their role in human neurons and AD has not yet been explored.

In this study, we used CRISPR/Cas9 genome engineering and human iPSC technologies to examine the relationship between CLU proteins and Aβ induced toxicity in human neurons in vitro. Given the importance of the ER-signal peptide in the generation of glycosylated, secreted CLU proteins in cells, we targeted this domain of CLU to prevent sCLU biogenesis. We generated human neurons lacking glycosylated CLU proteins, which displayed altered sensitivity to Aβ. We also used RNA-Seq analysis to identify gene pathways that may underly this altered sensitivity and contribute to our understanding of AD pathogenesis and identification of novel AD therapeutics.

## Materials and methods

### CRISPR/Cas9 gene editing to generate exon 2  −/− iPSC lines

For all work CTR M3 36S (CTR) iPSCs were used. The CTR iPSCs were previously generated from the Price lab at King’s College London from human keratinocytes obtained from a healthy, neurotypical adult male, as previously described^45–47^. CTR iPSCs were used to generate exon 2  −/− iPSCs (A4 and D1 clones), a dual single guide RNA (sgRNA) approach was chosen to recruit Cas9-2NLS (Synthego) to cut within intron 1 and 2 to remove *CLU* exon 2 (Supplementary Table 1)^48^. Transfection of CTR iPSCs was performed using Nucleofection and an optimised protocol (Synthego). Briefly, ribonucleotide protein complexes (RNPs) were assembled by mixing sgRNA and Cas9 in a 7.5:1 ratio: 3 µl (300 pmol) sgRNA was added to 2 µl (40 pmol) Cas9 in a total volume of 5 µl per well. CTR iPSCs were detached from wells using Accutase, and 500,000 cells were transferred to a falcon tube for transfection. Cells were centrifuged at 115*g* for 3 min and resuspended in 20 µl Lonza P3 Nucleofector™ solution (Lonza). 20 µl of cells were mixed with 5 µl of pre-prepared RNP complex solution and the total volume (25 µl) was transferred to a Nucleocuvette™ strip and placed into a 4D-X Core unit. Electroporation was performed using protocol ‘CA137’. Following transfection, cells were returned to a well of a 6-well plate into pre-warmed E8 containing 10 µM Rock inhibitor. Cells were incubated overnight, and media was replaced daily until cells reached 70–80% confluence and quality control assays were performed.

PCR amplification was used to confirm the homozygous knockout (KO) of *CLU* exon 2 in A4 and D1 iPSCs. For each clonal iPSC line, genomic DNA was extracted using the QuickExtract™ DNA Extraction Solution (Lucigen, QE09050) and was amplified using the AmpliTaq Gold™ 360 Master Mix according to manufacturer’s instructions (ThermoFisher, 4398881) with 200 nM forward and reverse primers (Supplementary Table 2). PCR amplifications were performed using the following parameters: 95 °C for 10 min, 95 °C for 30 s, 55 °C for 30 s, 72 °C for 45 s and a final extension at 72 °C for 7 min, a total of 39 cycles were used. PCR products were then used in agarose electrophoretic analysis by running on a 1% agarose gel and imaged using a BioRad Gel Doc (Supplementary Fig. 1). Following confirmation that A4 and D1 iPSCs were homozygous exon 2 KO clonal lines, quality control experiments were performed to check karyotype and pluripotency of the iPSC lines. Synthego provided karyotype data for A4 and D1 iPSCs, which were confirmed to be normal for both lines (Supplementary Fig. 2). For pluripotency analysis, A4 and D1 iPSCs were plated on 96-well plates, fixed, and stained with nuclear marker DAPI, and immunostaining for pluripotency markers OCT4 and SSEA4 to confirm the retention of pluripotency of A4 and D1 iPSCs following removal of *CLU* exon 2 (Supplementary Fig. 3).

### Neuronal differentiation

Human iPSCs were maintained in Essential 8 media (Life Technologies) on Geltrex coated plates (Nunc) until 100% confluent, at which point cells were differentiated according to a protocol adapted from Shi and colleagues^49,50^. iPSCs were cultured in 50% Neurobasal (Life Technologies) and 50% DMEM-F12 media (Life Technologies) supplemented with 1% N2 (Life Technologies), 2% B27 (Life Technologies), 1% Glutamax (Life Technologies), 10 µM SB-431542 (ApexBIO) and 1 µM LDN-193189 (Sigma) for 7 days with daily 100% media changes. Cells were dissociated with Accutase (Life Technologies) and replated on days 7, 12, 15 and 18 in 50% Neurobasal and 50% DMEM-F12 media supplemented with 1% N2 (Life Technologies), 2% B27 (Life Technologies) and 1% Glutamax (Life Technologies). On day 21, neural progenitor cells were replated on poly-ornithine and laminin coated plates at a density of 10,000 cells/well (96-well plate) and 200,000 cells/well (12-well plate) in B27 media (100% Neurobasal media supplemented with 2% B27 and 1% Glutamax). Media was changed after 24 h to remove cell debris. A full media change was performed on day 25 when 0.1 µM cytosine arabinoside (Sigma) was added to inhibit the growth of glial cells. A further full media change was performed 3 days later, and a half media change was performed another 3 days later. For all experiments, control and exon 2  −/− iPSCs were differentiated three independent times and neuronal identity was assessed by immunostaining (details of antibodies used can be found in Supplementary Table 3). The expression of neuronal marker MAP2 and cortical lineage marker CTIP2 was confirmed in all lines, and the numbers of MAP2 positive neurons and of CTIP2-positive MAP2 neurons were quantified to determine the percentage of neuronal cells in control and exon 2  −/− neuron cultures that were CTIP2 positive (Supplementary Fig. 4).

### Aβ_25–35_ oligomer preparation

Aβ_25–35_ Trifluoroacetate salt (BACHEM AG, Bubendorf, Switzerland, #4030205) was supplied at 5 mg with a purity of 97% (molecular weight 1060.28). Nuclease-free water was added to make up a solution of 2 mg/ml and stored at − 20 °C. Prior to use, aliquots were defrosted on ice at room temperature (RT). For measuring Aβ toxicity, three concentrations of Aβ_25–35_ (500 nM, 2 µM, 20 µM) was added to cells for 24 h prior to experimentation. For Western blotting and RNA-Seq, cells were treated for 24 h with 20 µM Aβ_25–35_ only.

### Western blotting

Prior to cell lysis, media samples were collected on ice and concentrated 45 times using Amicon Ultra-2 ml 10K Centrifugal Filter Units (Sigma, Aldrich, UFC201024) according to the manufacturer’s instructions. Protein content in the concentrated media samples was then measured following the addition of 150 µl Pierce 660 nm™ reagent (ThermoFisher, 22662). Protein content was measured relative to samples of Bovine Serum Albumin of known concentrations on a CLARIOstar Plus Plate reader (BMG LABTECH). For total cell lysates, 4 × 200 µl of laemmli buffer (BioRad) supplemented with HALT™ inhibitor (ThermoFisher, 78429) was added directly to cells. Lysates were collected, vortexed and boiled at 95 °C for 5 min at which point the supernatant was collected. For each sample, 4 µg of concentrated media was prepared in DPBS (Life Technologies, 14190094) and equal volumes of cell lysate and Laemmli buffer were separated by SDS-PAGE electrophoresis on 10% gels and transferred onto nitrocellulose membranes. The membranes were blocked with 5% BSA (made in 1 × TBST, tris-buffered saline and 0.1% Tween-20) for 1 h in agitation at RT. After blocking, membranes were incubated at 4 °C overnight in agitation with goat anti-clusterin primary antibody in 5% BSA (Santa Cruz, SC-6420, diluted 1:500). Membranes were then washed three times with 1 × TBST, incubated with secondary antibody for 2 h at RT in agitation (goat anti-donkey, IRDye^®^ 800 Li-COR Biosciences 926-32214, diluted 1:10,000 in 5% BSA). Membranes were again washed three times and imaged using an infrared scanner (LI-COR Biosystems). To confirm equal protein loading across samples, membranes were finally incubated with a rabbit anti-β-actin primary antibody (Cell Signalling Technology, D6A8, diluted in 1:1000 in 5% BSA) for 1 h at RT in agitation. Membranes were washed and incubated for 1 h at RT in agitation with a secondary antibody prior to imaging (donkey anti-rabbit, IRDye^®^ 680, Li-COR Biosciences 926-68073, diluted 1:10,000 in 5% BSA).

### Cell death assay

Cell death was quantified using the CytoTox-Glo Cytotoxicity Assay (Promega, G9291) according to manufacturer's instructions. For all treatment conditions, cell death was compared relative to neurons that were untreated for 24 h. Following Aβ_25–35_ treatment (500 nM, 2 µM, 20 µM) 50 μl AAF-Glo was added to each well for 15 min. Luminescence as a result of apoptosis induced loss of membrane integrity was read on a PHERAstar FSX Microplate reader (BMG LABTECH) giving a measure of cell death induced by Aβ_25–35_ treatment. Then, 50 μl lysis reagent containing digitonin and AAF-Glo was added to wells for 15 min. The addition of these reagents was used to induce death in remaining live cells in each well, quantified as the total dead count. Luminescence was again recorded and was used as a measure of the total number of cells in each well. Dead/Total Dead Ratios were then calculated and compared to the ratios of untreated neuron cultures; an increase in this ratio following treatment with Aβ_25–35_ would reflect an increase in the number of dead cells in the culture indicating Aβ_25–35_ treatment had caused significant increases in apoptosis and cell death.

### Neurite length assay

To quantify neurite damage following Aβ_25–35_ treatment (500 nM, 2 µM, 20 µM), neurons were fixed with 4% paraformaldehyde-DPBS at RT for 15 min. Cells were washed with DPBS three times prior to immunostaining. Cells were simultaneously permeabilised and blocked in DPBS containing 0.25% Triton-X and 10% donkey serum (Life Technologies) for 1 h at RT. Chicken anti-MAP2 (Abcam, ab5392, 1:1000) primary antibodies were diluted in DPBS containing 0.25% Triton-X and 5% donkey serum. Cells were washed three times with TBST and incubated for 1 h at RT in secondary antibody goat anti-chicken 647 diluted in blocking solution (Life Technologies, A-21449, 1:500). Cells were then washed once with Hoechst 33342 for 30 s (Thermo Fisher, 0.01 μg/ml diluted in DPBS). Cells were then washed a further three times with DPBS prior to imaging on an Opera Phenix High Content Screening System (Perkin Elmer) under 20 × magnification. Immunofluorescent images were analysed using the Harmony High-Content Analysis Software (Perkin Elmer) using a neurite length assay developed in the analysis interface. First, intact nuclei were identified following positive staining by Hoechst. Neurites protruding from cell bodies stained positive for MAP2 in the 647 channel were then traced and quantified.

### RNA-Seq and analysis

For RNA-Seq experiments, neurons were treated with 20 µM Aβ_25–35_ for 24 h. Total RNA was collected using the Qiagen RNeasy Mini PLUS Kit (Qiagen) according to the manufacturer’s instructions from untreated and Aβ_25–35_ treated control and exon 2  −/− neurons from three independent differentiations. 100 ng of RNA were used for library preparation and analysis (Wellcome Trust Centre for Human Genetics, University of Oxford). Only samples with an RNA integrity number greater than 7 were used for subsequent sequencing. TruSeq RNA Library Preparation Kit (Illumina) was used, and mRNA was enriched by polyA selection. The mRNA fraction was selected from total RNA and converted to cDNA. cDNA was then end83 repaired, A-tailed and adaptor ligated. Samples were sequenced at 150 paired ends on a NovaSeq6000 for PolyA-RNA-Seq. We conducted quality control (QC) of the RNA-seq data using FastQC (version 0.11.5)^51^, for annotation of low-quality adapters and reads followed by trimming in Cutadapt (version 1.13)^52^, and then aligned by BWA-MEM (version 0.7.15)^53^ to the human reference genome GRCh37.EBVB95-8wt.ERCC. The average sequencing quality of all reads across samples was above 36 (Sanger scording, > Q30). Reads that overlap multiple features were resolved and summarised to the gene-level using the ‘summarizeOverlaps’ function of the (GenomicAlignments R package)^54^, with the follow settings: (i) mode = Union, (ii) singleEnd = F, (iii) ignore.strand = T, (iv) fragments = T and features were summarised to the gene-level. This yielded an Ensembl gene ID by sample counting matrix of the class S4 (R-object). The latter counting matrix was modelled using the DESeq2 pipeline which performs (i) size factors estimation, (ii) dispersion estimation and (iii) Gamma-Poisson Generalized Linear Model (GLM) fitting^55^. We report differentially expressed (DE) genes based on log2 fold-change (abs(FC) > 1) and p-values (p < 0.05) adjusted with Benjamini–Hochberg false discovery rate (FDR). Over-representation analysis (ORA) of ontology and pathway terms performed with hypergeometric tests (clusterProfiler, PMID:22455463)^56^ using gene sets encompassing Gene Ontology (GO) subsets such as biological process (BP), molecular function (MF), and cellular component (CC). Also, incorporating biological pathways gene sets from the MSigDB Collections (version c2.cp.kegg.v7.5.1, BROAD Institute)^57,58^ and topological signalling pathway enrichment implemented in SPIA^59^. Dimension reduction techniques such as Principal Component analysis (PCA) and the Uniform Manifold Approximation and Projection (UMAP) were applied to the normalised counts across all samples. The normalised counts were mean-centred and standardized, then dimension reduction with PCA, and afterwards using as input the first 20 principal components (PC) for the computation of UMAP^60^.

### Statistical analysis

**Figure 1:**
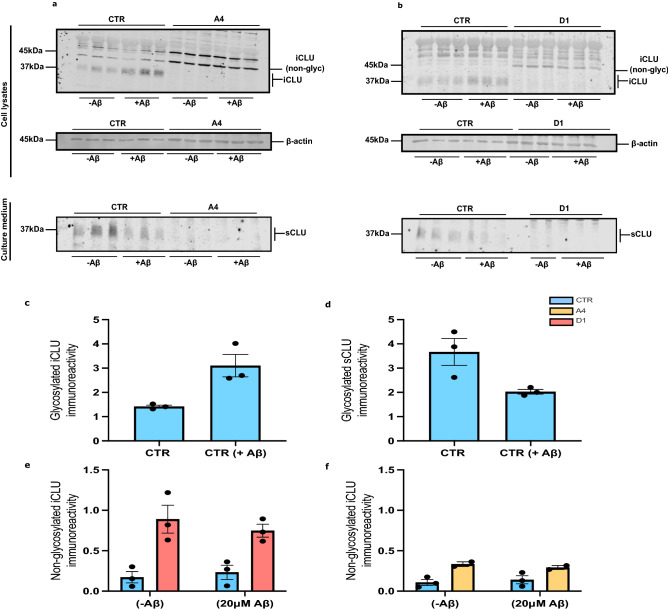
Aβ_25–35_ treatment alters trafficking of glycosylated CLU in human neurons. CTR and exon 2  −/− neurons (A4 and D1) were treated for 24 h with 20 μM Aβ_25–35_. Concentrated media and total cell lysate samples were collected, ran on a western blot and immunostained for CLU. (**a**) Western blot analysis of clusterin expression in cell lysate and culture media of untreated and Aβ_25–35_ treated CTR and A4 neurons (**b**) and untreated and Aβ_25–35_ treated CTR and D1 neurons. In both (**a**) and (**b**) β-actin were used as a loading control for protein loading of cell lysate samples. The absence of β-actin expression was confirmed in all culture media samples. (**c**,**d**) CLU immunoreactivity in cell lysates was normalised to β-actin immunoreactivity for each sample individually. This was then normalised to the immunoreactivity of the untreated CTR samples. (**c**) Intracellular, glycosylated CLU immunoreactivity was significantly increased (p = 0.0221*) while (**d**) secreted, glycosylated CLU was significantly decreased in CTR neurons (p = 0.0432*) following treatment with Aβ_25–35_. Exon 2  −/− neurons were demonstrated to not express glycosylated CLU proteins, either intracellularly or secreted into the media. (**e**,**f**) Loss of CLU exon 2 increased immunoreactivity of non-glycosylated CLU proteins in exon 2  −/− neurons at basal conditions (A4: p = 0.0077** and D1: p = 0.0348*) but Aβ_25–35_ did not alter the immunoreactivity of non-glycosylated, intracellular CLU species in either CTR or exon 2  −/− neurons (CTR: p = 0.09785 and A4: p = 0.7991, CTR: 0.9255 and D1: p = 0.9154). Immunoreactivity is reported as mean ± standard error of the mean (SEM). Mean values were calculated from neuronal samples collected from three independent differentiations of neurons, of which each contained triplicate samples (n = 3). Two-Way ANOVAs were performed for all comparisons (*p < 0.05, **p < 0.01, ***p < 0.001 and ****p < 0.0001). Western blot images used in this figure have been cropped for clarity, all original blots are presented in Supplementary Fig. 5.

**Figure 2:**
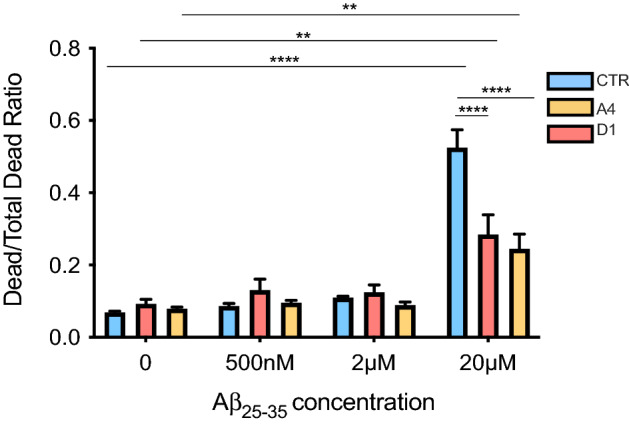
Exon 2  −/− neurons exhibit lower cell death rates when treated with Aβ_25–35_. CTR and exon 2  −/− neurons were treated with a one of three concentrations of Aβ_25–35_ (500 nM, 2 μM or 20 μM). After 24 h of treatment, cell death induced by Aβ_25–35_ was quantified. AAF-Glo reagent was added to cultures to measure protease activity released from apoptotic cells, giving a measurement of death induced by Aβ_25–35_ treatment. Death was then induced in the remaining, live cells by addition of digitonin and AAF-Glo. Death was quantified again giving a measurement of total number of death cells in each culture, representing the total number of cells. For each condition, the Dead/Total Dead Ratio was calculated and compared. Before Aβ_25–35_ treatment, the ratios of exon 2  −/− and CTR neurons were comparable (p > 0.9999 for all comparisons). 20 μM Aβ_25–35_ significantly increased the Dead/Total Dead Ratios of both CTR and exon 2  −/− neurons (CTR; p < 0.0001****, A4; p = 0.0200* and D1; p = 0.0254*. The percentage increase in number ratios for each cell line: CTR: 658.67% increase, A4: 208.24% increase and D1: 209.61% increase). 500 nM and 2 μM Aβ_25–35_ treatments did not induce significant cell death in any culture. Dead/Total Dead Ratios of 20 μM Aβ_25–35_ treated CTR cultures were significantly higher than the Ratios demonstrated in exon 2  −/− cultures (20 μM Aβ_25–35_ CTR vs A4: p < 0.0001**** and 20 μM Aβ_25–35_ CTR vs D1: p < 0.0001****) but were similar between the exon 2  −/− cultures (A4 vs D1: p = 0.9952). This reflects less cell death quantified in exon 2  −/− cultures than in CTR neurons. Dead/Total Dead Ratios are reported as mean ± SEM and Two-Way ANOVAs were performed for all concentrations (*p < 0.05, **p < 0.01, ***p < 0.001 and ****p < 0.0001). Mean values were calculated from samples collected from three independent differentiations of neurons (n = 3), of which each contained 6 replicant samples.

**Figure 3:**
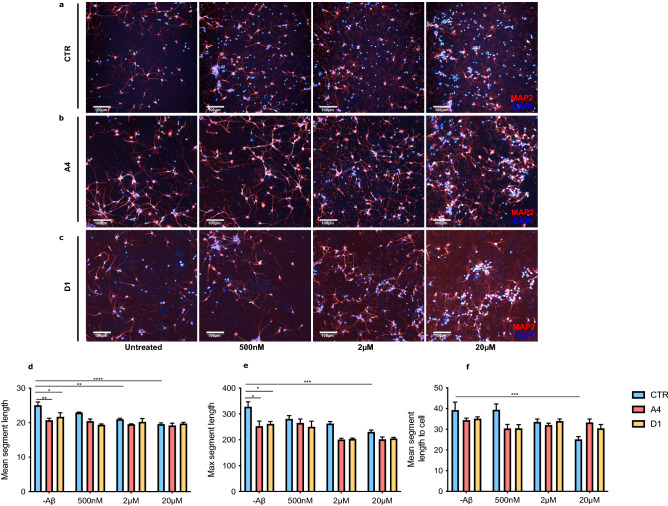
Aβ_25–35_ does not induce neurite retraction in exon 2  −/− neurons. CTR and exon 2  −/− (A4 and D1) neurons were treated for 24 h with one of three concentrations of Aβ_25–35_. Following treatment, neurons were fixed, immunostained for DAPI (blue) and MAP2 (red) and imaged. Representative images of (**a**) CTR, (**b**) A4 and (**c**) D1 neurons. (**d**–**f**) Changes in length of neuronal processes were then quantified using an automated analysis software pipeline that identified MAP2 positive areas. Before treatment, mean segment length (CTR vs A4: p = 0.0310** and CTR vs D1: p = 0.0379*) and maximum segment length were significantly reduced in exon 2  −/− neurons than CTR neurons (CTR vs A4: p = 0.0161* and CTR vs D1: p = 0.0487*). 20 μM Aβ_25–35_ induced significant reductions in neurite length parameters in CTR neurons but not in exon 2  −/− neurons (CTR: mean segment length; p < 0.0001****, maximum segment length; p = 0.0009***, mean segment length to cell; p = 0.0010***). Exon 2  −/− neurons did not display evidence of Aβ_25–35_ induced neurite damage at any concentration of Aβ_25–35_. Values are reported as mean ± SEM and Two-Way ANOVAs were performed for all time points (*p < 0.05, **p < 0.01, ***p < 0.001 and ****p < 0.0001). Mean values were calculated from samples collected from three independent differentiations of neurons (n = 3), of which each contained 6 replicant samples.

Statistical significance was set as p < 0.05 for all assays and experiments used in this study. Statistical tests were performed using GraphPad Prism (v. 7.02). A normal distribution in cell populations was assumed and two-tailed parametric tests were used. For experiments examining changes in CLU protein abundance in control neurons, analysis was performed using an unpaired *t* test (Fig. 1). For comparisons of CLU protein abundance between untreated and Aβ treated control and exon 2  −/− neurons analysis was performed by One-way ANOVA with Tukey’s Multiple Comparisons post hoc test (Fig. 1). Additionally, One-way ANOVA with Tukey’s Multiple Comparisons post hoc test was used to analyse differences in CTIP2 expression in control, A4 and D1 neurons (Supplementary Fig. 4d,e). For comparisons of changes in cell death and neurite length between untreated and Aβ_25–35_ treated control, A4 and D1 neurons, analysis was performed using a Two-way ANOVA with Tukey’s multiple comparisons post hoc test (Figs. 2 and 3). In analysis where post hoc analysis was performed adjusted p values are reported. Data are shown as mean ± standard error of the mean (SEM) unless otherwise indicated.

## Results

### Glycosylated CLU protein trafficking is altered by Aβ_25–35_ in human neurons

While Aβ_1–42_ is considered to the pathological Aβ species its use in vitro is impaired due to its instability when used in an aggregated fibrillary state^61^. Numerous studies have demonstrated similarities in activity in vitro between Aβ_25–35_ and Aβ_1–42_^62–64^, making Aβ_25–35_ a suitable alternative for use in this work. A variety of concentrations of Aβ_25–35_ have been demonstrated to induce cell death previously^35,65,66^. Given our group has demonstrated significant alterations in CLU trafficking in rodent neurons treated with Aβ_25–35_^35^ our first aim was to determine if Aβ_25–35_ treatment alters trafficking of glycosylated CLU in human neurons. Control (CTR) and exon 2  −/− neurons were treated with 20 μM Aβ_25–35_ for 24 h, after which media and cell lysates were collected, ran on a western blot and immunostained for CLU. The abundance of CLU proteins was first quantified in all samples separately and normalised to the abundance of β-actin for all samples. Finally, this was then normalised to CTR samples to compare abundance of CLU proteins in exon 2  −/− neurons to that of CTR neurons. Thus, we have compared CLU levels between treated and untreated neurons, as well as between exon 2  −/− and CTR neurons to examine the effect of both, Aβ_25–35_ and the loss of *CLU* exon 2.

Exon 2  −/− neurons did not express glycosylated CLU proteins, confirming previous reports that *CLU* exon 2 is required for the generation of mature, cleaved and glycosylated CLU proteins (Fig. 1a,b, full-length blots for CLU, both intracellular and secreted, are provided in Supplementary Fig. 5. Raw image files β-actin are also provided, these have not been altered/cropped, full-length blots for β-actin were not taken originally and thus, cannot be provided in all cases). To examine if Aβ_25–35_ altered glycosylated CLU trafficking, we compared levels of sCLU and intracellular (iCLU) clusterin protein expression between untreated and 20 μM Aβ_25–35_ treated CTR neurons and showed that intracellular CLU was significantly increased (Fig. 1a–c, p = 0.0221*) while secretion of CLU was significantly decreased (Fig. 1a,b,d, p = 0.0432*, original blots are presented in Supplementary Fig. 5). This response was absent in exon 2  −/− neurons. This is the first demonstration that Aβ_25–35_ exposure alters CLU trafficking in human neurons and supports previous studies that demonstrated CLU trafficking is altered in a variety of cell types/lines including rodent neurons, U251, SH-SY5Y, N2a and LNCaP cell lines following the induction of several stressors including Aβ_25–35_ treatment and ER stress (induced by MG132 proteasome inhibitor)^35,36,38,40^.

The translation of *CLU* variant 1 Δ exon 2 mRNA generates intracellular CLU proteins that are neither cleaved nor glycosylated, due to the absence of the ER-signal peptide^41,43,67^. Abundance of these CLU proteins is increased following stress^43^, and is thought to facilitate cell death^42,43^. Therefore, we next aimed to examine whether Aβ_25–35_ treatment induces an upregulation of these proteins in human neurons. First, abundance of non-glycosylated CLU proteins was compared between CTR and exon 2  −/− neurons prior to treatment with Aβ_25–35_. Unsurprisingly, abundance of non-glycosylated CLU proteins was significantly higher in exon 2  −/− neurons than in CTR neurons following the removal of *CLU* exon 2 (Fig. 1a,e,f). Next, the effect of Aβ_25–35_ treatment on non-glycosylated CLU abundance was examined. 20 μM Aβ_25–35_ did not alter the abundance of non-glycosylated CLU in either CTR or exon 2  −/− neurons (Fig. 1a,e,f). This contrasts with previous research that demonstrated that stress in cancer cell lines induces an upregulation of non-glycosylated CLU proteins. Our data show that stress induced by Aβ_25–35_ treatment in human neurons has a specific effect on the trafficking of glycosylated CLU proteins, but not on the overall abundance of the non-glycosylated CLU proteins.

### Exon 2  −/− neurons display less Aβ_25–35_ induced cell death than CTR neurons

Having demonstrated that Aβ_25–35_ induces alterations in CLU trafficking in CTR neurons but does not alter abundance of non-glycosylated CLU, we next aimed to determine if this resulted in altered sensitivity of exon 2  −/− neuron to Aβ_25–35_. To do this, we first treated CTR and exon 2  −/− neurons with increasing concentrations of Aβ_25–35_ for 24 h (500 nM, 2 μM or 20 μM, respectively). After treatment, cell death was quantified by luminescence using the CytoTox-Glo Cytotoxicity Assay (Fig. 2).

Previous studies have identified a potential role for non-glycosylated CLU in facilitating cellular response to stress and apoptosis^42,43^. Additionally, overexpression of non-glycosylated CLU has been demonstrated to sensitise cells to stress^42^. Therefore, we aimed to determine if the loss of *CLU* exon 2 and the increased abundance of non-glycosylated CLU proteins at basal conditions in exon 2  −/− neurons altered their sensitivity to Aβ_25–35_ induced cell death. Dead/Total Dead Ratios were compared across all treatment conditions and between genotypes to determine if exon 2  −/− neurons were more sensitive to Aβ_25–35_ induced cell death (Fig. 2). In untreated conditions, Dead/Total Dead Ratios of exon 2  −/− neurons did not significantly differ to that of the CTR neurons, indicating that the proportion of dead neurons at basal conditions was not influenced by the genotype. No significant increase in ratios was observed at either 500 nM or 2 μM Aβ_25–35_ treatment for either CTR or exon 2  −/− neurons, indicating that these concentrations were not sufficient to induce a significant increase in cell death. However, a significant increase in ratios, reflecting a significant increase in the number of dead cells, was observed using 20 μM Aβ_25–35_ treated neuron cultures. This was observed in both, CTR and exon 2  −/− neuron cultures. Interestingly higher amounts of cell death were quantified in CTR cultures treated with 20 μM Aβ_25–35_ compared to exon 2  −/− cultures, since the Dead/Total Dead ratios of the latter where significantly lower. This finding indicates significantly fewer exon 2  −/− neurons underwent cell death because of Aβ_25–35_ treatment. These data demonstrate that the loss of *CLU* exon 2 and the increased abundance of non-glycosylated CLU proteins in human neurons reduces sensitivity to Aβ_25–35_.

### Exon 2  −/− neurons are protected against Aβ_25–35_ induced neurite retraction

The observed reduction in Aβ_25–35_ induced cell death in exon 2  −/− neurons suggests that the lack of glycosylated CLU proteins renders neurons less sensitive to Aβ_25–35_ induced toxicity. To further explore this, we examined Aβ_25–35_ induced neurite retraction by measuring changes in neurite length in CTR and exon 2  −/− neurons.

As before, CTR and exon 2  −/− neurons were treated with one of three concentrations of Aβ_25–35_. After 24 h, neurons were fixed, immunostained and imaged. Neurons that were positively stained for nuclear marker DAPI and neuronal marker MAP2 were imaged (Fig. 3a–c) and an automated pipeline was used to identify MAP2 positive neurite processes and quantify their length. Neurite length parameters were quantified and compared between genotypes and Aβ_25–35_ treatment conditions (Fig. 3d–f).

Previously, we demonstrated that cell death prior to treatment was comparable between CTR and exon 2  −/− neurons, indicating that loss of *CLU* exon 2 did not impact neuronal death in the absence of an Aβ challenge. Accordingly, we aimed to determine if this was similar in the case of neurite length. Surprisingly, two neurite length parameters measured were significantly reduced in exon 2  −/− neurons prior to treatment with Aβ_25–35_, suggesting that loss of *CLU* exon 2 may impact neurite length even at basal conditions.

Next, we aimed to determine what concentration, if any, is sufficient to induce neurite damage in human neurons resulting in significant neurite retraction. For all parameters, 20 μM Aβ_25–35_ consistently caused a significant reduction in neurite length in CTR neurons, no significant changes in neurite length were identified in CTR neurons treated with 500 nM or 2 μM Aβ_25–35_, except in the case of mean segment length, which was significantly reduced in CTR neurons treated with 2 μM Aβ_25–35_. Finally, we explored whether the observed changes in neurite length in CTR neurons could also be detected in exon 2  −/− neurons. Interestingly, exon 2  −/− neurons did not demonstrate significant neurite retraction at any concentration of Aβ_25–35_, although did demonstrate reduced neurite length in basal conditions. Taken together, our data show that exon 2  −/− neurons are protected against both, Aβ_25–35_ induced neurite retraction and Aβ_25–35_ induced neuronal death.

### CLU exon 2 removal downregulates extracellular matrix (ECM) pathways

**Figure 4:**
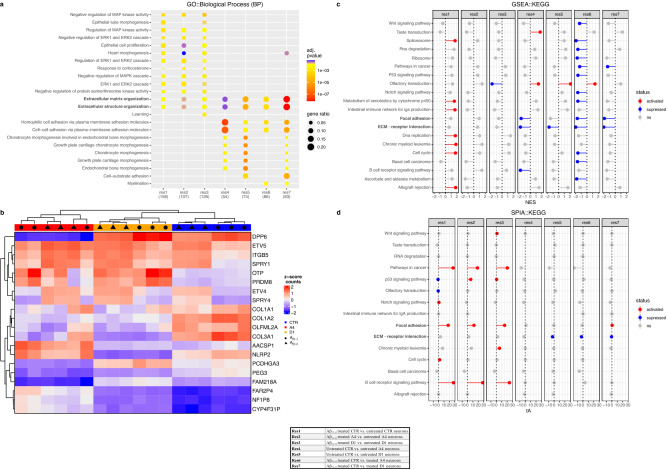
Extracellular matrix (ECM) genes and pathways are downregulated in exon 2  −/− neurons. RNA-Seq analysis of exon 2  −/− and CTR neurons reveal suppression of ECM related genes and pathways in exon 2  −/− neurons. (**a**) GO (biological process) enrichment analysis reveal downregulation of collagen and ECM genes in exon 2  −/− neurons (res 4 and 5). (**b**) Heatmap visualisation of top 20 most variable genes clustered with complete hierarchical clustering using z-scored counts. Column annotations represent Aβ_25–35_ treatment condition and neuron genotype (CTR, A4 or D1). (**c**) Functional directionality analysis identified focal adhesion and ECM receptor interaction were suppressed in exon 2  −/− neurons, which was not altered by Aβ_25–35_ treatment. (**d**) Topology identified a significant suppression of ECM-receptor interaction and PI3K-Akt signalling pathways were suppressed in exon 2  −/− neurons, again unaltered by Aβ_25–35_. Figure legend outlines comparisons made in this analysis: res1; Aβ_25–35_ treated vs untreated CTR neurons, res2; Aβ_25–35_ treated vs untreated A4 neurons, res3; Aβ_25–35_ treated vs untreated D1 neurons, res4; untreated A4 vs untreated CTR neurons, res5; untreated D1 vs untreated CTR neurons, res6; Aβ_25–35_ treated A4 vs CTR neurons and res7; Aβ_25–35_ treated D1 vs CTR neurons. Differential gene expression satisfied thresholds for both fold change (> 1.5) and adjusted p value (< 0.05) were included in this analysis. Numbers of each res represent the number of differentially expressed genes enriched in each pathway for each comparison. Gene set enrichment satisfied adjusted p value (< 0.05). NES (enrichment score normalised to mean enrichment of random samples of the same size). *tA* the observed total perturbation accumulation in the pathway, *pPERT* probability to observe a total accumulation more extreme than tA only by chance. pPERT* < 0.05, ** < 0.01 and *** < 0.001.

Having identified differences in the responses of exon 2  −/− and CTR neurons to Aβ_25–35_ treatment, we aimed to use RNA-Seq analysis to identify changes in gene expression that may contribute to the altered sensitivity of exon 2  −/− neurons to Aβ_25–35_ induced toxicity. Differences in gene expression were compared between CTR and exon 2  −/− neurons and between untreated and Aβ_25–35_ treated neurons giving a total of 7 comparisons (Table 1 and Supplementary Table 4). UMAP analysis revealed distinct cell cultured based on treatment condition for CTR, A4 and D1 neurons (untreated vs Aβ_25–35_), additionally, separation of cell clusters based on neuronal genotype (CTR vs A4/D1) were observed with A4 and D1 counts clustering more closely to each other than with CTR counts (Supplementary Fig. 6). Differentially expressed genes in each comparison were grouped according to GO pathway enrichment to identify significantly altered pathways (Fig. 4a). Ten pathways were differentially activated between exon 2  −/− and CTR neurons in basal conditions prior to treatment with Aβ_25–35_ (res 4 and 5). Genes implicated in extracellular matrix (ECM) organisation, extracellular structure organisation, homophilic cell adhesion via plasma membrane adhesion molecules, cell–cell adhesion via plasma membrane adhesion molecules and several chondrocyte morphogenesis pathways were all were downregulated in exon 2  −/− neurons. Several ECM implicated genes were identified in a heatmap displaying the top 20 most highly variable genes (Fig. 4b), including *COL1A1*, *COL1A2*, *COL3A1* and *PCDHGA3*. These were demonstrated to be more highly expressed in CTR neurons than in A4 and D1 neurons, implicating a depression of ECM-related gene expression. Functional enrichment analysis was performed (Supplementary Table 5). Functional directionality analysis of KEGG pathway enrichment (Fig. 4c) identified focal adhesion and extracellular matrix receptor interaction pathways and pointed to a general downregulation of ECM in exon 2  −/− neurons. Importantly, this was unaltered by Aβ_25–35_ in exon 2  −/− neurons. Topology analysis performed on KEGG pathway enrichment data supported these findings (Fig. 4d) and we could identify a suppression of ECM receptor interaction in exon 2  −/− neurons independent of treatment condition. Additionally, this analysis identified suppression of PI3K-Akt signalling in exon 2  −/− neurons, also independent of the treatment condition. These data suggest that loss of exon 2 and the subsequent alterations in CLU localisation in human neurons may result in reduced expression of genes encoding proteins that perform functions in the ECM and in the PI3K-Akt pathway. These findings indicate that the loss of CLU exon 2 and the subsequent changes in CLU localisation is sufficient to reduce sensitivity of human neurons in vitro to Aβ_25–35_ induced toxicity and downregulate genes performing ECM functions. It is unclear as to whether this reduced sensitivity is attributed to the loss of glycosylated CLU proteins or the observed increase in non-glycosylated intracellular CLU proteins in exon 2  −/− neurons.

**Table 1:** A list of RNA-Seq comparisons.

Comparison	Genotypes and conditions compared
Res1	Aβ_25–35_ treated CTR vs. untreated CTR neurons
Res2	Aβ_25–35_ treated A4 vs. untreated A4 neurons
Res3	Aβ_25–35_ treated D1 vs. untreated D1 neurons
Res4	Untreated CTR vs. untreated A4 neurons
Res5	Untreated CTR vs. untreated D1 neurons
Res6	Aβ_25–35_ treated CTR vs. treated A4 neurons
Res7	Aβ_25–35_ treated CTR vs. treated D1 neurons

Differences in gene expression were analysed between CTR, A4 and D1 neurons, both in untreated and Aβ_25–35_ treated neurons. The effect of Aβ_25–35_ treatment on gene expression was examined in res 1–3 comparisons, while the effect of CLU exon 2 KO was examined in res 4–7.

### Identification of gene expression changes induced by Aβ_25–35_ treatment

Having identified transcriptomic changes arising in neurons following removal of *CLU* exon 2, we next used our RNA-Seq dataset to identify changes in gene expression arising in neurons following treatment with Aβ_25–35_ that may be regulating neurite damage and cell death induced by Aβ_25–35_. Using topology-based analysis of KEGG pathway changes, several pathways were found altered in both, CTR and exon 2  −/− neurons, including apoptosis (activated), MAPK signalling (activated), p53 signalling (activated), cholinergic synapse (activated), glutamatergic synapse (inhibited) and axon guidance (inhibited) (Table 2 and Supplementary Fig. 7). Given these pathways were regulated by Aβ_25–35_ in CTR and exon 2  −/− neurons, it is likely that these contribute to cell death observed in both lines. For example, it is reasonable to suggest the observed activation in p53 signalling, apoptosis and MAPK signalling are related to Aβ_25–35_ induced cell death in both, exon 2  −/− and CTR neurons. However, it is not clear why cell death is induced by exon 2  −/− neurons but only to a lesser extent than in CTR neurons. Interestingly, although neurite retraction was absent in Aβ_25–35_ treated exon 2  −/− neurons, like in CTR neurons, axon guidance and glutamatergic synapse pathways were inhibited while cholinergic synapses were activated. These observations would be predicted in CTR neurons displaying neurite damage but since neurite retraction is absent in exon 2  −/− neuron it remains unclear why these pathways appear altered. We hypothesise that this could be due to differences in the extent of activation and inhibition of the pathways highlighted by our analysis in CTR and exon 2  −/− neurons, which may be reflected in the reduced sensitivity of exon 2  −/− neurons to cell death and neurite retraction; this is something to be explored in further studies. Therefore, examination of levels of activation and inhibition of pathways including axon guidance and cholinergic and glutamatergic synapses will help identify if they play a crucial role in mediating Aβ_25–35_ induced neurite retraction.

**Table 2:** A list of pathways altered by Aβ_25–35_ in human neurons.

Pathways activated by Aβ_25–35_	Pathways suppressed by Aβ_25–35_	Cell lines affected
Apoptosis, MAPK signalling, p53 signalling, cholinergic synapse	Glutamatergic synapse, axon guidance	CTR and exon 2 −/−
Cell cycle, complement and coagulation cascades	LTD	CTR only

Pathways significantly altered (activated/suppressed) by Aβ_25–35_ treatment in CTR and exon 2  −/− neurons, or CTR neurons alone.

Finally, no pathway was identified as significantly altered in both A4 and D1 neurons that were not altered in CTR neurons following Aβ_25–35_ treatment. This suggests that exon 2  −/− neurons’ altered sensitivity to Aβ_25–35_ induced toxicity may arise due to an absence of pathway activation/inhibition observed in CTR neurons. As supporting evidence, three further pathways were altered in CTR neurons and not in exon 2  −/− neurons. Cell cycle and complement and coagulation cascades were all significantly activated in CTR neurons following Aβ_25–35_ treatment, while long term depression (LTD) was suppressed (Supplementary Fig. 7). This could be an indication that one or more of these pathways plays a crucial role in mediating Aβ_25–35_ induced neurite retraction, given that activation of these pathways was not observed in exon 2  −/− neurons in response to Aβ_25–35_ treatment.

## Discussion

CLU has long been implicated in AD, however, its exact role in AD pathogenesis and disease development remains unclear. Our study demonstrates a central role for CLU proteins in facilitating Aβ_25–35_ induced cell death and neurite damage in human neurons in vitro.

Previous studies have examined the cytoprotective role of secreted CLU and the pro-apoptotic role of non-glycosylated CLU proteins^43,68–70^. However, these studies have focussed on the role of CLU proteins in cancer pathways in immortal cancer cell lines and few studies have directly manipulated the expression of non-glycosylated CLU proteins in human neurons to explore their importance in neurodegeneration. In contrast to previous work, we demonstrate that stress induced by Aβ_25–35_ does not induce increased expression of non-glycosylated CLU in human neurons. Therefore, the previously identified pro-apoptotic role of non-glycosylated CLU proteins may not be universal and may be dependent on factors such as the type of cell and source of stress. Yang et al., demonstrated that overexpression of non-glycosylated CLU proteins in cancer cells sensitizes cells to stress and induces more death in basal conditions^42^. This is not observed in human neurons where we have demonstrated that exon 2  −/− neurons displayed similar levels of cell death in basal conditions, indicating that increased expression of non-glycosylated CLU did not result in increased neuronal death, although changes to basal neurite length were demonstrated in these same cells. Further examination of the role of non-glycosylated CLU species may reveal their role in altering basal neurite length in exon 2  −/− neurons and if this is contributing to the reduced sensitivity of these neurons to Aβ_25–35_ or if there is a masking of the effect induced by Aβ_25–35_ on neurite length due to the reduction in basal conditions. It would therefore be valuable to assess the effect of *CLU* exon 2 removal on neuron development in vitro; the neurons used in this research are developmentally immature and thus, culturing for longer periods of time would be beneficial to compare the expression of neurons maturity markers and synapse formation in CTR and exon 2  −/− neurons. This would provide clarity on the development of neurons lacking glycosylated CLU and its effect on neuronal sensitivity to Aβ_25–35_. To the best of our knowledge, the authors are unaware of any study identifying CLU or Aβ_25–35_ as a regulator of neuron maturity.

We cannot rule out a combinatory effect on Aβ_25–35_ sensitivity reflected by both the loss of glycosylated CLU and increase in non-glycosylated CLU proteins in the same neurons. Very little is known of the role of non-glycosylated CLU proteins in cells, especially in human neurons, it is suggested that non-glycosylated CLU proteins are induced by stress and apoptosis^41–43^, however, these studies failed to categorically show that these CLU proteins are needed before cell death can occur, or their increased expression arises following the induction and initiation of apoptosis. Indeed, there may be a role for the non-glycosylated CLU proteins in neurite development that is influencing their sensitivity to Aβ_25–35_ induced toxicity. Although others have suggested a role of non-glycosylated CLU proteins generated from *CLU* mRNA lacking exon 2, this has only been demonstrated in immortal cancer cell lines using overexpression studies^41–43^, we are the first to generate iPSCs and iPSC-neurons lacking exon 2 and increased non-glycosylated CLU proteins in the absence of non-glycosylated CLU proteins. It is reasonable to suggest that the role of these proteins will likely differ between cell types, especially in cancer derived lines and neurons and is likely influenced by the mechanism of the stressor.

Exon 2  −/− iPSC-neurons displayed reduced sensitivity to Aβ_25–35_ induced cell death and neurite damage, suggesting that it is the glycosylated version of CLU that facilitate Aβ_25–35_ toxicity. We have therefore demonstrated that non-glycosylated CLU proteins likely do not play a critical role in mediating neurodegeneration in human neurons induced by Aβ_25–35_.

Although typically considered a secreted protein, glycosylated CLU proteins have been shown to localise within cells^35,36,40^, however, the mechanisms leading to intracellular retention of sCLU have not been described. Numerous studies have demonstrated that trafficking of glycosylated CLU proteins is altered by stress in a variety of cell types/lines^36–41^, resulting in reduced secretion and increased intracellular retention of CLU. Additionally, *CLU*-AD mutations and Aβ_25–35_ treatment of rodent neurons have been shown to alter CLU trafficking, providing a potential mechanistic role of CLU in mediating neurodegeneration^17,35^.

To date, few studies have attempted to dissect whether stress induced alterations in CLU trafficking act to facilitate cell stress or act to protect cells from further damage. Gregory et al. demonstrated altered CLU trafficking by ER stress in N2a cells resulting in increased binding of intracellular CLU and TDP-43, which reduced both the aggregation and toxicity of TDP-43, thus suggesting altered CLU trafficking in this context provides a protection to cells^40^. In comparison, previous work by our group found evidence that altered CLU trafficking facilitates Aβ_25–35_ induced cell death in rodent neurons and that Aβ_25–35_ increased intracellular CLU retention and *CLU* knockdown provided protection from Aβ_25–35_ induced neurodegeneration^35^. It is therefore likely that stress-induced CLU trafficking alterations and subsequent increases in intracellular CLU as well as reduced secretion can be both protective and detrimental to cells dependent on the type of stress and cell. Understanding the importance of glycosylated CLU proteins is particularly important since several studies have identified plasma CLU as a promising marker for AD^71^; higher plasma CLU levels are associated with increased hippocampal atrophy and increased rate of clinical progression^72,73^.

**Figure 5:**
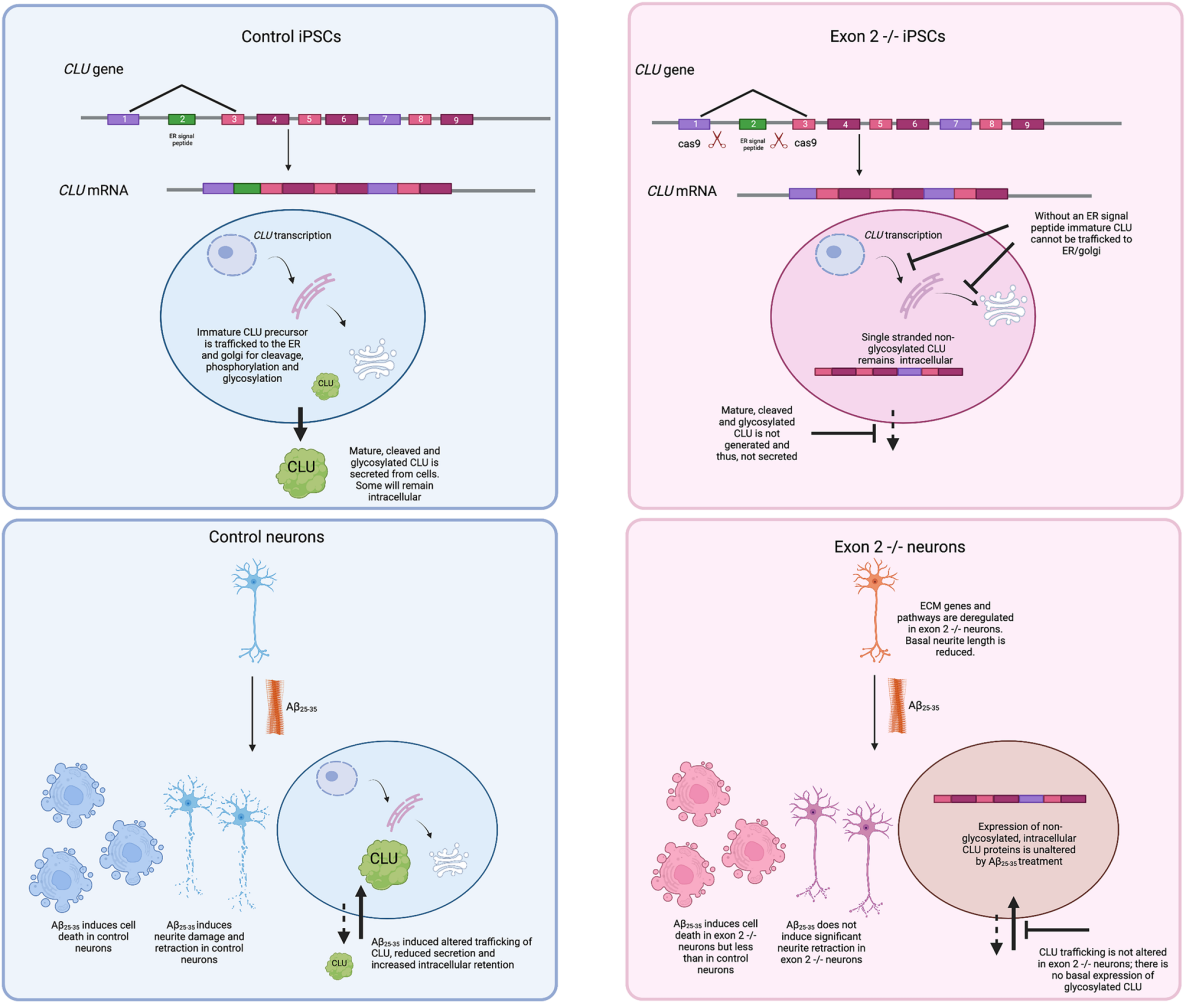
Summary diagram. Graphic illustration highlighting key results observed in this study. To alter the type and localisation of CLU proteins generated by iPSCs CRISPR/Cas9 gene editing was used; iPSCs lacking CLU exon 2 do not produce mature, glycosylated and cleaved CLU proteins and therefore do not secrete CLU proteins. Instead, exon 2  −/− iPSCs generate single polypeptide CLU protein that does not undergo glycosylated and remains intracellular. Following differentiation of CTR and exon 2  −/− iPSCs into neurons, neurons were treated with Aβ_25–35_ and phenotypes compared to assess if altered CLU protein production alters the sensitivity of neurons to Aβ_25–35_ induced toxicity. Aβ_25–35_ treatment induced significant cell death and neurite damage to control neurons, this was accompanied by an alteration in CLU trafficking resulting in increased intracellular retention and reduced secretion of glycosylated CLU proteins. CLU trafficking was not altered in exon 2  −/− neurons since they do not express glycosylated CLU proteins. In contrast to previous reports, no evidence was found that non-glycosylated CLU protein abundance is increased following induction of stress or cell death. Exon 2  −/− neurons displayed significant cell death following Aβ_25–35_ treatment but no significant neurite damage was observed. We suggest that the partial protection displayed by exon 2  −/− neurons may be attributed to the altered expression and activity in extracellular matrix genes and pathways as highlighted by RNA-seq analysis. Diagram created with BioRender.com.

Our data clearly show that loss of glycosylated CLU protein in human neurons in vitro provides partial protection against Aβ_25–35_ induced toxicity, which we postulate may arise from ECM remodelling (Fig. 5). Although cell death was still induced by Aβ_25–35_ in exon 2  −/− neurons, it was significantly less than control neurons and neurite damage was absent in exon 2  −/− neurons. What remains unclear is whether the observed protection arises because of the loss of intracellular glycosylated CLU proteins or the loss of secreted glycosylated CLU proteins, or both. Although previous studies have suggested a pro-apoptotic role of non-glycosylated CLU proteins in cells^42,43^, little is really known of their physiological and pathological functions in cells. We demonstrate an increase in the abundance of non-glycosylated CLU proteins in exon 2  −/− neurons, which is not altered by Aβ_25–35_ treatment. It would be crucial to determine if protection observed in exon 2  −/− is conferred by the increased abundance of these CLU proteins.

It is therefore crucial to determine the roles played by intracellular and secreted CLU proteins in Aβ_25–35_ induced toxicity to determine the exact roles played by CLU proteins in Aβ_25–35_ induced cell death and neurite retraction and the underlying mechanisms. Several studies have shown that CLU proteins bind directly to Aβ peptides^20,37^ to regulate Aβ uptake and clearance by astrocytes^37^. This role is attributed to secreted glycosylated CLU proteins. We hypothesise that in control neurons in response to Aβ_25–35_ treatment, secreted CLU is taken back into cells, potentially as a method of protecting cells. However, this role may facilitate toxicity if concentrations of Aβ_25–35_ peptides are high in the cellular media. sCLU proteins may bind to Aβ_25–35_ peptides and facilitate the uptake of CLU-Aβ_25–35_ complexes into neurons, thereby promoting entry of Aβ_25–35_ peptides into cells. Once inside the cell, Aβ_25–35_ peptides can then induce cytotoxicity^74–77^. Thus, in this context CLU is facilitating Aβ_25–35_ toxicity. Further interrogation of this relationship is required. Several receptors for CLU have been identified, including the megalin receptor^21,34^, but this interaction is not well described and has not been analysed in iPSC-neurons. In exon 2  −/− neurons, the absence of secreted CLU may result in reduced Aβ_25–35_ uptake into neurons, resulting in a lower concentration of Aβ_25–35_ peptides accumulating in the neurons and thus, less cell damage. Secreted clusterin has been shown to bind Aβ peptides and may mediate the internalisation and clearance of Aβ following binding to cell surface receptors^34,37,78,79^. Clusterin has been demonstrated to facilitate the uptake of Aβ by astrocytes^37^ but others have demonstrated clusterin may hamper Aβ uptake^80,81^. This interaction so far has not been explored in human neurons, and the model generated by our group would facilitate this; CTR and exon 2  −/− neurons could be treated with Aβ by addition to the media and imaging could then be used to measure Aβ uptake in both sets of neurons, to determine if glycosylated CLU proteins are facilitating Aβ uptake into neurons. Since we described only partial protection in exon 2  −/− neurons, other mechanisms must also facilitate the uptake of Aβ_25–35_ peptides and Aβ_25–35_ toxicity in human neurons, in addition to CLU proteins.

Interestingly, our RNAseq data has identified only three pathways that were altered by Aβ_25–35_ treatment in CTR and not in exon 2  −/− neurons: cell cycle, complement and coagulation cascades and LTD. The absence of response in these pathways in exon 2  −/− neurons suggest a role for CLU in these pathways, exploration of the role of these pathways in mediating Aβ_25–35_ toxicity in vitro may aid in understanding CLU role in facilitating Aβ_25–35_ neurotoxicity but also the role of these individual pathways in contributing to both cell death and neurite damage induced by Aβ_25–35_. It would be interesting to determine if the targeting of these pathways in CTR neurons to prevent their activation (in the case of cell cycle and complement/coagulation cascades) and prevent their suppression (LTD) would manifest as a reduction in Aβ_25–35_ induced phenotypes like those demonstrated in our exon 2  −/− neurons. The complement system is heavily implicated in AD and although it may not initiate disease pathology it is considered to be a driver of neurodegeneration^82^. Additionally, clusterin is a key inhibitor of the complement system^34,83^. Further exploration of the effect CLU exon 2 knockout has on the complement system in exon 2  −/− neurons will be invaluable in evaluating the source of their partial protection to Aβ_25–35_. The complement system however, is complex and it may be that certain complement components are driving pathology while others are protective. Nevertheless, our data implicate a key role of complement activation in mediating Aβ_25–35_ toxicity in vitro*.* Additionally, exon 2  −/− knockout resulted in a suppression of the PI3K-Akt pathway, a known regulator of GSK-3β; activation of Akt inhibits GSK-3β and thus, downstream reduces tau hyperphosphorylation^84^. The suppression of PI3K-Akt in exon 2  −/− neurons may thus, have effects in vitro beyond Aβ induced cell death and neurite damage; it would be interesting to determine if the suppression of PI3K-Akt has any effect on tau phosphorylation and synapse integrity following Aβ treatment in exon 2  −/− neurons.

Changes to the ECM were demonstrated in exon 2  −/− neurons and we postulate that ECM remodelling contributes to the partial protection observed in exon 2  −/− neurons to Aβ_25–35_ providing evidence for targeting the ECM as a therapeutic option for AD and neurodegeneration. The ECM is composed of a range of diverse proteins that serve a multitude of functions^85^. Those include regulation of cell death and survival^86^, albeit this is not clearly described in neurons, synaptic function^87,88^ and neurite outgrowth^89,90^, all three of which are significantly altered in AD and by Aβ_25–35_. ECM remodelling is associated with several diseases including AD^91–94^. Numerous ECM proteins have been described as upregulated or downregulated in AD brains^94^. For example, Collagen V and fibronectin are upregulated in the cerebral cortex of AD patients in early stages of disease progression and may arise from a reduction in proteolytic activity and may contribute to early changes in the AD brain^95^. Several ECM proteins play a crucial role in regulating synaptic transmission and thus, changes to these proteins may result in a disruption in synaptic function and result in abnormal synaptic activity, and impaired learning and memory^96,97^. ECM proteins have also been shown to alter Aβ toxicity. Heparan and chondroitin sulfate proteoglycans bind to Aβ peptides reducing Aβ_25–35_ induced toxicity in hippocampal cultures and PC12 cells. Indeed, reducing the expression of chondroitin sulfate proteoglycans enhances Aβ_1–42_ toxicity in neurons^98^. Other ECM proteins have been shown to promote Aβ aggregation and plaque formation^99,100^. Most recently, a large scale deep multi-layer analysis study of AD brains identified the ECM to strongly correlate with AD traits^101^, providing strength to our observations that the ECM may play a key role in mediating Aβ_25–35_ induced toxicity in human neurons. More work needs to be done to fully understand the role of the ECM in AD since both, protective and facilitatory roles have been identified. In our study it is apparent that the ECM directly contributes to toxicity induced by Aβ_25–35_ in vitro in human iPSC-neurons since ECM remodelling is associated with neuroprotective properties to iPSC-neurons. Given the varied functions of ECM proteins in neurons, it is essential to identify the key proteins that are central to the partial protection observed in exon 2  −/− neurons to determine if they can be utilised as a therapeutic target to provide neuroprotection.

## Conclusions

Our data show that glycosylated CLU proteins facilitate neurotoxicity induced by Aβ_25–35_ in human neurons. In fact, we demonstrate a role for CLU in mediating both Aβ_25–35_ induced neurite retraction and cell death; the absence of glycosylated CLU and increased non-glycosylated CLU proteins provided partial protection against Aβ_25–35_ to neurons in vitro. Previous research has identified the role of non-glycosylated CLU proteins in promoting cell death and increasing cell sensitivity to stress. However, these studies had been carried out in cancer cell lines and not neurons. Our work clearly shows the importance of cellular context and clearly demonstrates that, in neurons, non-glycosylated CLU does not promote neuronal degeneration but that increased expression results in a protection against neurite loss and neuronal death. We have identified several genes and pathways that potentially underlie this neuroprotection including those that may play a critical role in regulating cell death and neurite damage induced by Aβ_25–35_. Our data will facilitate the identification of key components underlying Aβ_25–35_ toxicity and identify potential therapeutic targets to prevent neurodegeneration in AD.

## Supplementary Information

Supplementary Figure 1.

Supplementary Figure 2.

Supplementary Figure 3.

Supplementary Figure 4.

Supplementary Figure 5.

Supplementary Figure 6.

Supplementary Figure 7.

Supplementary Table 1.

Supplementary Table 2.

Supplementary Table 3.

Supplementary Table 4.

Supplementary Table 5.

## Data Availability

The RNA-Seq datasets generated and analysed during this current study are available in the NCBI GEO repository (GSE207466), which can be accessed here: https://www.ncbi.nlm.nih.gov/geo/query/acc.cgi?acc=GSE207466). For data, please contact corresponding author Professor Noel Buckley.
