# Supplementary material for: Glycosylated clusterin species facilitate Aβ toxicity in human neurons
Source: Sci Rep. 2022 Nov 3;12:18639. doi: 10.1038/s41598-022-23167-z (PMC9633591; doi:10.1038/s41598-022-23167-z)
Supplement: Supplementary file 1 — Supplementary Figure 1. [file 41598_2022_23167_MOESM1_ESM.pdf]

a

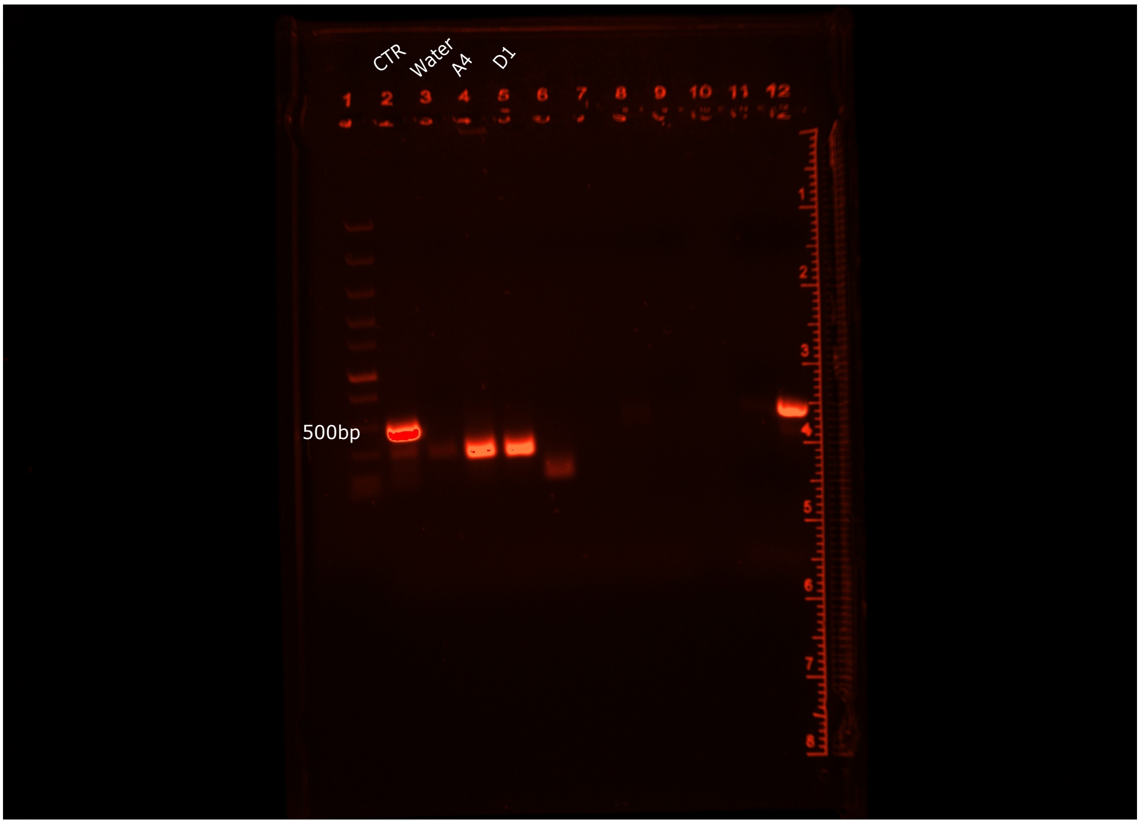

b

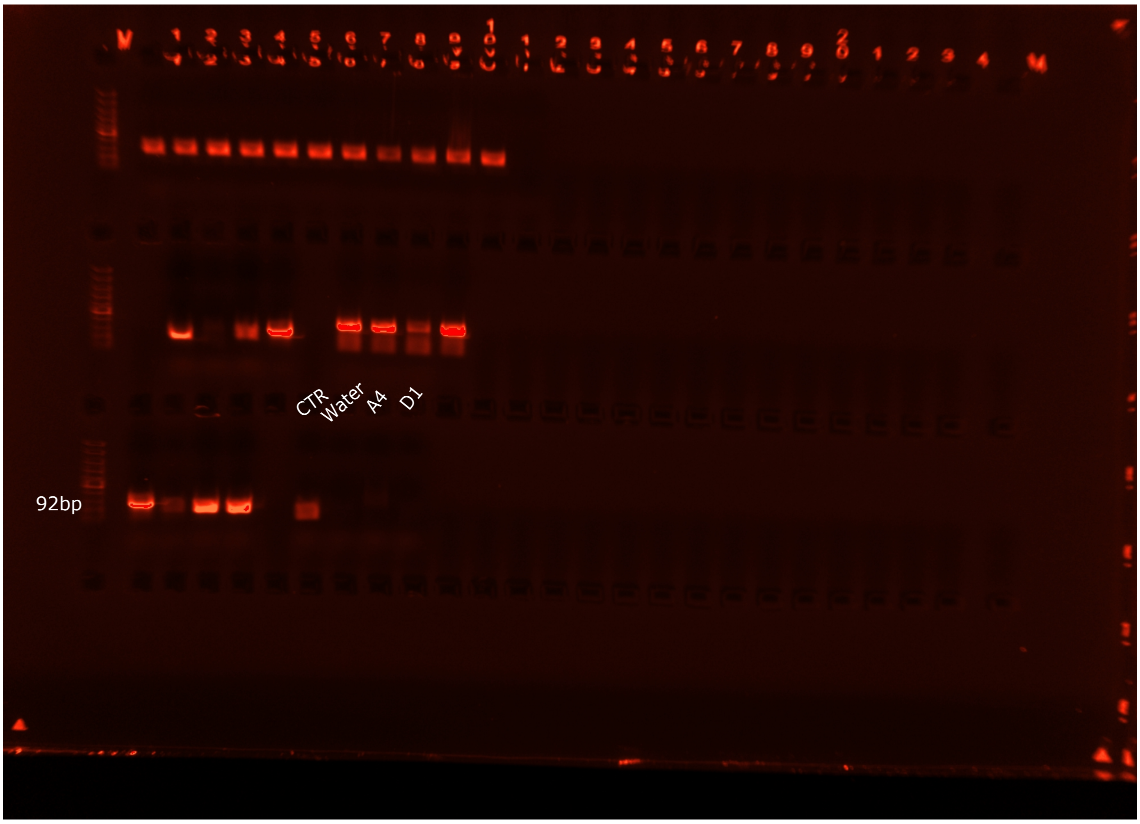

Supplementary figure 1. Agarose gel electrophoresis to confirm removal of CLU exon 2 from human iPSCs. (a) Primers positioned within introns 1 and 2 were initially used to confirm deletion of exon 2 from A4 and D1, indicating by the presence of a PCR product of smaller size in both these lanes (4 and 5) compared to CTR (lane 2). (b) Primers positioned within exon 2 were then used to confirm the removal of exon 2 from both alleles of A4 and D1 iPSCs (lanes 7 and 8, lower panel) compared to CTR (lane 5)
