# Supplementary material for: Glycosylated clusterin species facilitate Aβ toxicity in human neurons
Source: Sci Rep. 2022 Nov 3;12:18639. doi: 10.1038/s41598-022-23167-z (PMC9633591; doi:10.1038/s41598-022-23167-z)
Supplement: Supplementary file 2 — Supplementary Figure 2. [file 41598_2022_23167_MOESM2_ESM.pdf]

Supplementary figure 2

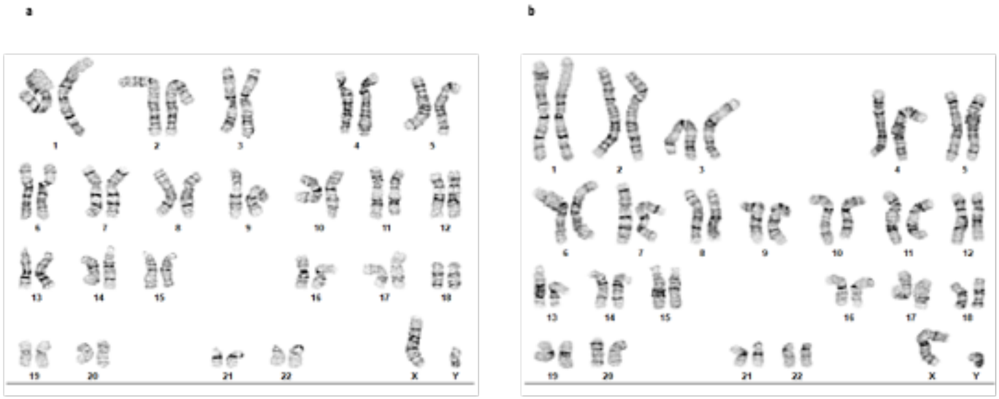

Supplementary figure 2. Karyotyping of (a) A4 and (b) D1 iPSCs. No chromosomal abnormalities were identified in either line. A4 (46, X, Y) and D1 (46, X, Y). A standard metaphase karyotype (band resolution 475-525) was performed and identified no missing or additional chromosomes in either line.
