# Supplementary material for: Glycosylated clusterin species facilitate Aβ toxicity in human neurons
Source: Sci Rep. 2022 Nov 3;12:18639. doi: 10.1038/s41598-022-23167-z (PMC9633591; doi:10.1038/s41598-022-23167-z)
Supplement: Supplementary file 3 — Supplementary Figure 3. [file 41598_2022_23167_MOESM3_ESM.pdf]

Supplementary figure 3

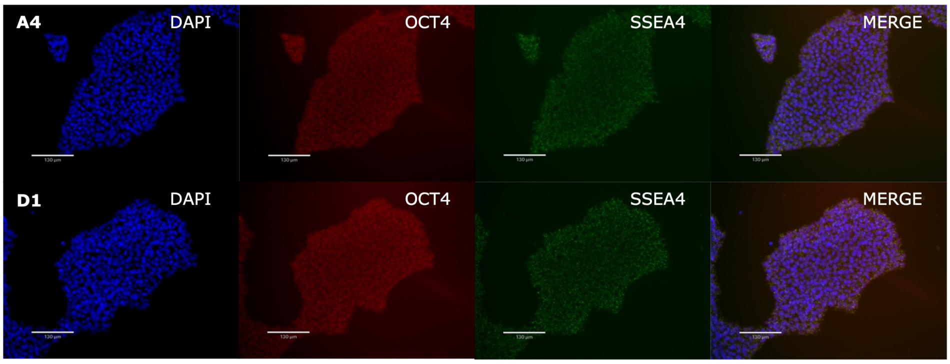

Supplementary figure 3. Immunostaining of A4 and D1 iPSCs to confirm expression of pluripotency markers OCT4 (red) and SSEA4 (green). Expression was confirmed for both markers in A4 and D1 iPSCs and was confirmed to be nuclear (DAPI, blue) due to co-localisation with DAPI (purple).
