# Supplementary material for: Glycosylated clusterin species facilitate Aβ toxicity in human neurons
Source: Sci Rep. 2022 Nov 3;12:18639. doi: 10.1038/s41598-022-23167-z (PMC9633591; doi:10.1038/s41598-022-23167-z)
Supplement: Supplementary file 4 — Supplementary Figure 4. [file 41598_2022_23167_MOESM4_ESM.pdf]

Supplementary figure 4

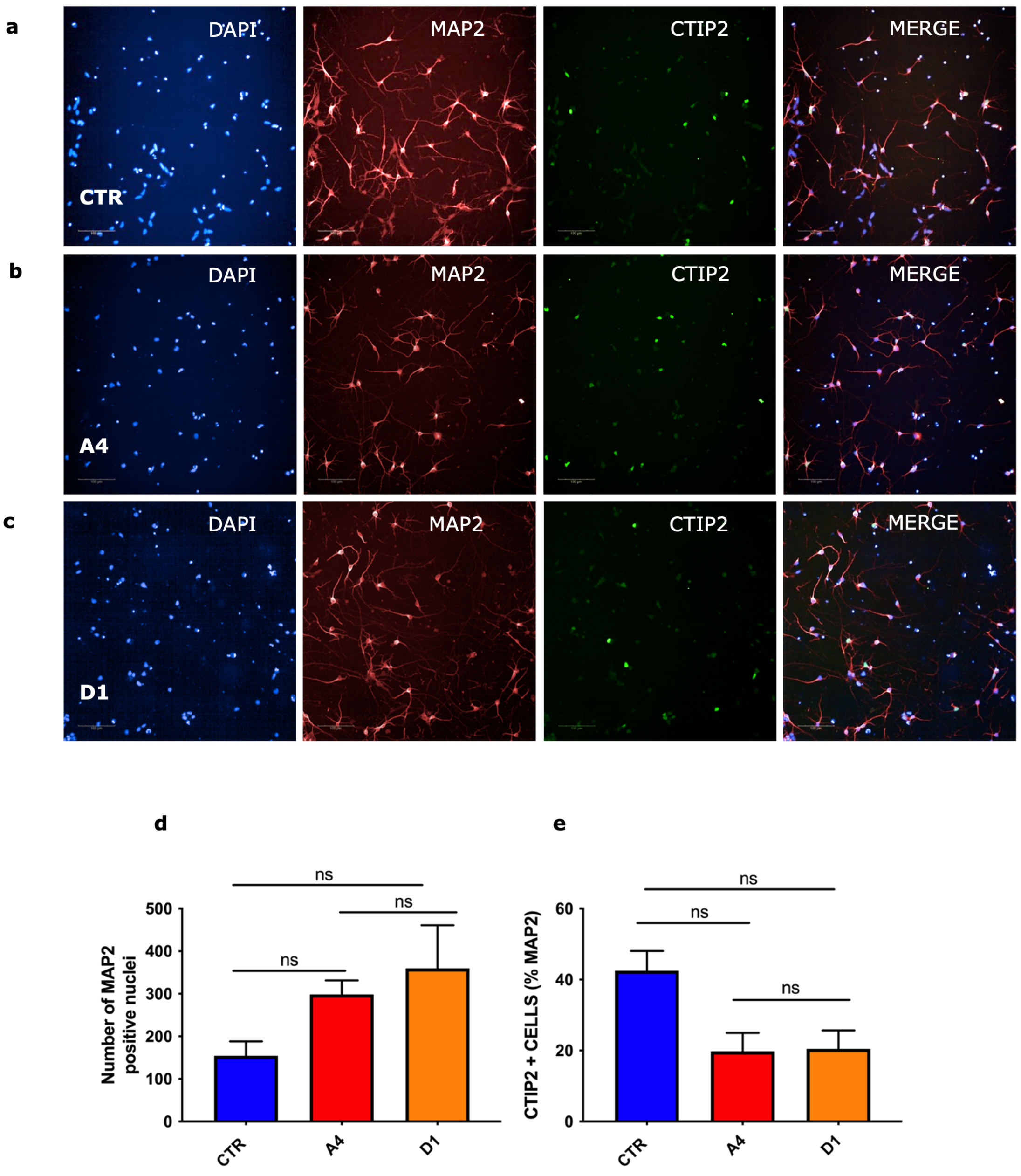

Supplementary figure 4. Immunostaining of (**a**) CTR, (**b**) A4 and (**c**) D1 iPSC-neurons to confirm neuronal identity (MAP2) and cortical lineage (CTIP2). (**d**) The number of MAP2 positive neurons was similar between genotypes (p=0.1474; CTR vs. A4: p=0.3227, CTR vs. D1: p=0.1396, A4 vs. D1: p=0.7873). (**e**) The percentage of CTIP2 positive MAP2 neurons was significantly influenced by neuron genotype (p=0.0387*), however, no significant differences were revealed following multiple comparisons testing (CTR vs. A4: 0.0699, CTR vs. D1: p=0.0656 and A4 vs. D1: p=0.9957). The percentage of CTIP2

positive MAP2 neurons for all cultures were: CTR: 42.5 ± 5.6%, A4: 19.8 ± 5.2%, and D1: 20.5 ± 5.2%). Data are shown as mean ± SEM. Mean values were calculated from neuronal samples collected from three independent differentiations of neurons, of which each contained triplicate samples (n=3). Significance was tested using One-Way ANOVA with Tukey's Multiple comparisons analysis $P<0.05$*.
