# Supplementary material for: Glycosylated clusterin species facilitate Aβ toxicity in human neurons
Source: Sci Rep. 2022 Nov 3;12:18639. doi: 10.1038/s41598-022-23167-z (PMC9633591; doi:10.1038/s41598-022-23167-z)
Supplement: Supplementary file 5 — Supplementary Figure 5. [file 41598_2022_23167_MOESM5_ESM.pdf]

Supplementary figure 5

Figure 1a upper blot iCLU original

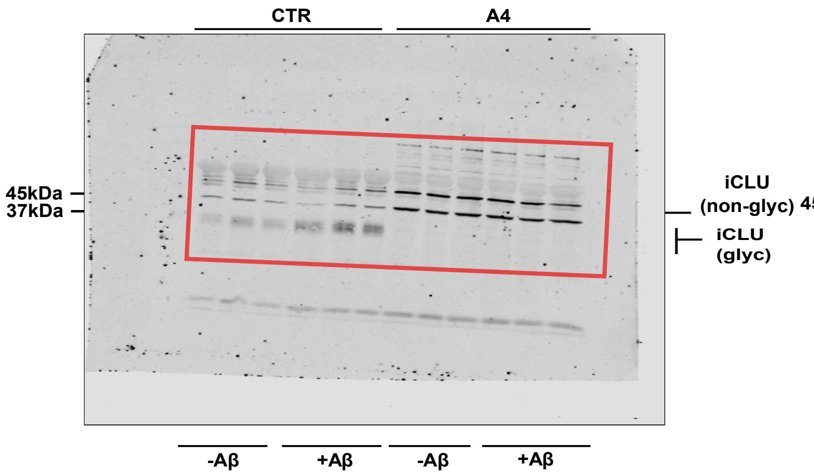

Figure 1a beta actin blot original

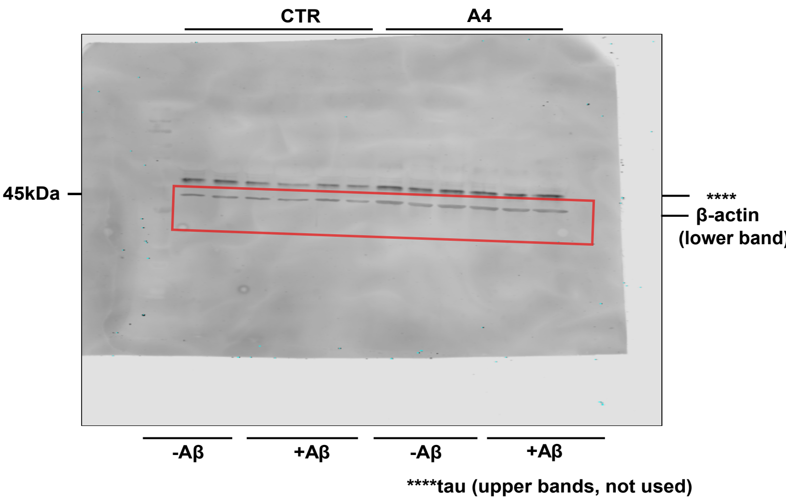

Figure 1b upper blot iCLU original

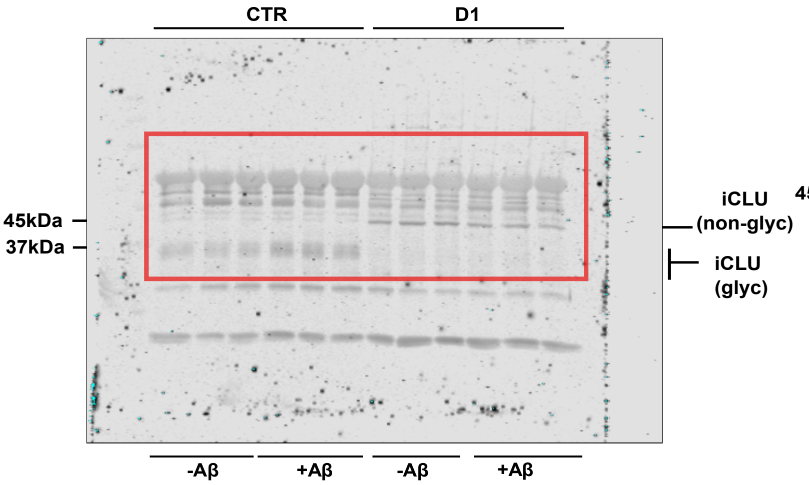

Figure 1b beta actin blot original

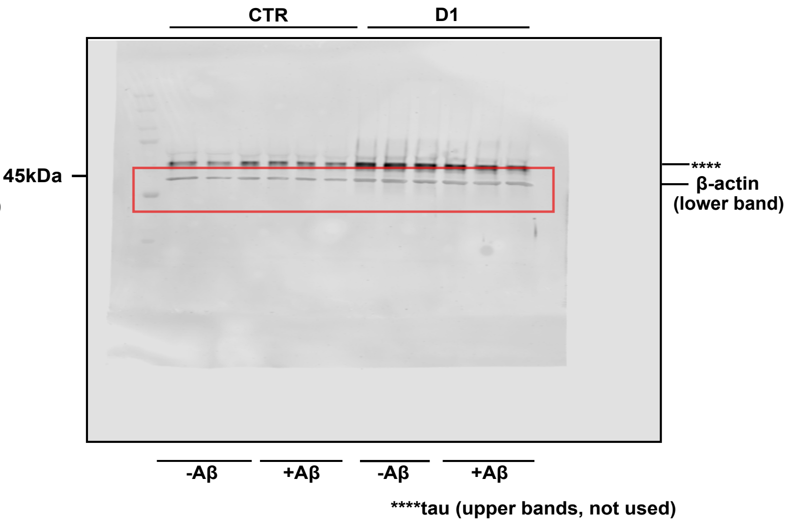

Figure 1a lower blot sCLU original blot

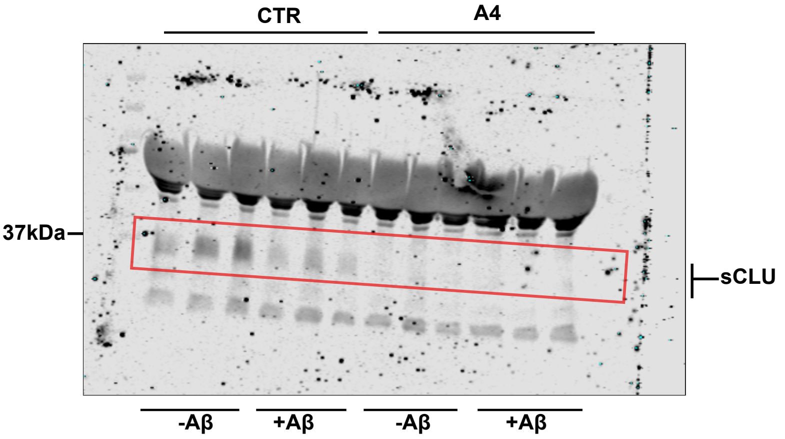

Figure 1b lower blot sCLU original blot

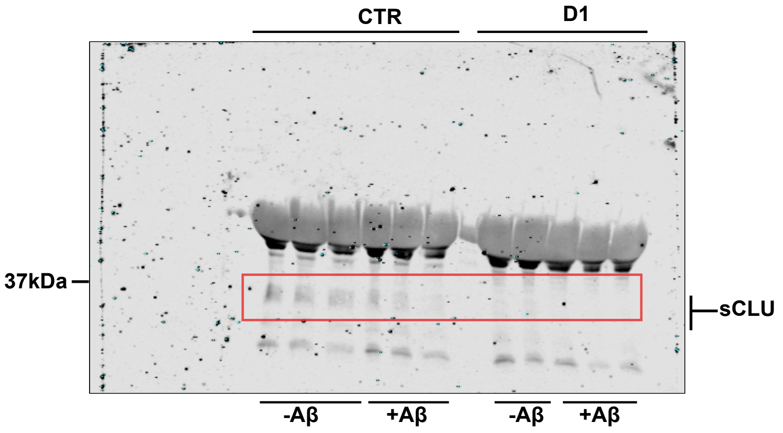

Supplementary figure 5. Original and uncropped western blots for immunostaining of CLU and β-actin.

All blots were stained with goat anti-clusterin primary to stain both intracellular and secreted clusterin proteins. For loading control staining blots were stained with rabbit anti- β-actin primary.

Blots displaying β-actin also display additional bands staining for tau (rabbit primary) but these were not used in subsequent analysis
