# Supplementary material for: Glycosylated clusterin species facilitate Aβ toxicity in human neurons
Source: Sci Rep. 2022 Nov 3;12:18639. doi: 10.1038/s41598-022-23167-z (PMC9633591; doi:10.1038/s41598-022-23167-z)
Supplement: Supplementary file 6 — Supplementary Figure 6. [file 41598_2022_23167_MOESM6_ESM.pdf]

Supplementary figure 6

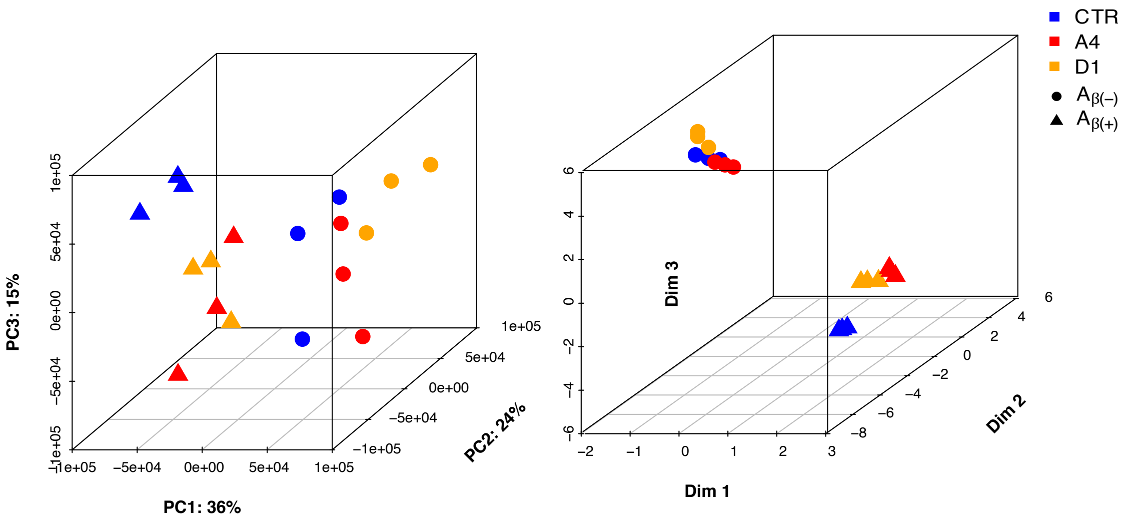

Supplementary figure 6. Dimensionality reduction of the normalized counts across samples. Principal component analysis (PCA) accounting for 75% (PC1-PC3) of the cumulative variance of uniform manifold approximation and projection (UMAP).
