# Supplementary material for: Glycosylated clusterin species facilitate Aβ toxicity in human neurons
Source: Sci Rep. 2022 Nov 3;12:18639. doi: 10.1038/s41598-022-23167-z (PMC9633591; doi:10.1038/s41598-022-23167-z)
Supplement: Supplementary file 7 — Supplementary Figure 7. [file 41598_2022_23167_MOESM7_ESM.pdf]

Supplementary figure 7

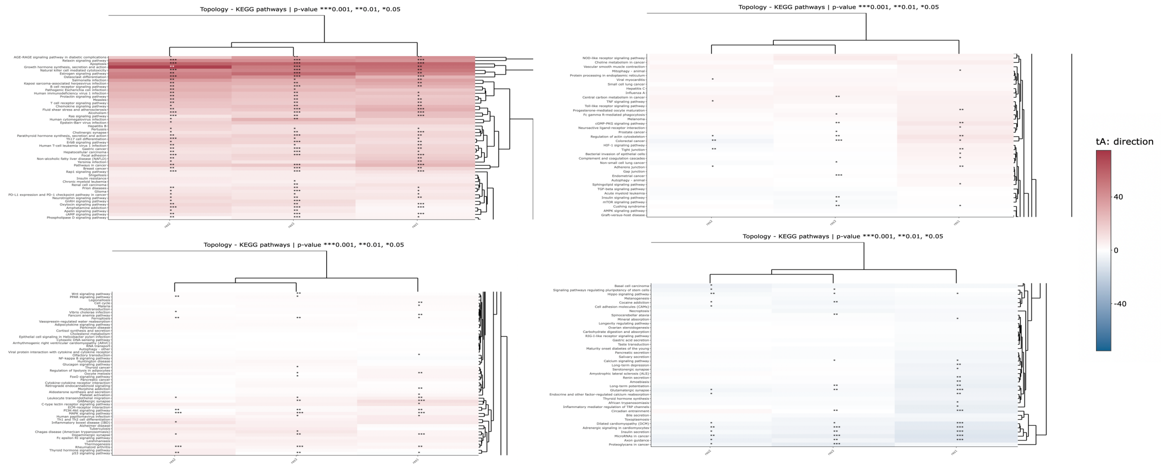

Supplementary figure 7. Topology analysis of KEGG pathway enrichment was performed to identify pathways activated or suppressed by A$\beta_{25\text{-}35}$ treatment in CTR and exon 2 -/- neurons (res 1, 2 and 3). Several relevant pathways were activated by A$\beta_{25\text{-}35}$ treatment including apoptosis, p53, MAPK signalling, cholinergic synapses in neurons of both genotypes. Fewer relevant pathways were inhibited by A$\beta_{25\text{-}35}$ treatment including glutamatergic synapses and axon guidance in neurons of both genotypes. Three pathways altered by A$\beta_{25\text{-}35}$ treatment only in CTR neurons were identified: cell cycle (activated), long term depression (inhibited) and complement and coagulation regulation (activated). Comparisons: res1: untreated CTR vs A$\beta_{25\text{-}35}$ treated CTR iPSC-neurons, res2: untreated A4 vs A$\beta_{25\text{-}35}$ treated A4 iPSC-neurons and res3: untreated D1 vs A$\beta_{25\text{-}35}$ treated D1 iPSC-neurons. tA: the observed total perturbation accumulation in the pathway, pPERT: probability to observe a total accumulation more extreme than tA only by chance. pPERT*<0.05, **<0.01 and ***<0.001
