# Supplementary material for: Glycosylated clusterin species facilitate Aβ toxicity in human neurons
Source: Sci Rep. 2022 Nov 3;12:18639. doi: 10.1038/s41598-022-23167-z (PMC9633591; doi:10.1038/s41598-022-23167-z)
Supplement: Supplementary file 8 — Supplementary Table 1. [file 41598_2022_23167_MOESM8_ESM.docx]

**Supplementary table 1: Guide RNA sequences used in this study.**

5’ sgRNA ACTACCAGAGGACAAGAGAC

3’ sgRNA GTACTACAAGGAGACCGGTG
