# Supplementary material for: Glycosylated clusterin species facilitate Aβ toxicity in human neurons
Source: Sci Rep. 2022 Nov 3;12:18639. doi: 10.1038/s41598-022-23167-z (PMC9633591; doi:10.1038/s41598-022-23167-z)
Supplement: Supplementary file 9 — Supplementary Table 2. [file 41598_2022_23167_MOESM9_ESM.docx]

**Supplementary table 2: List of PCR primers used in this study to confirm exon 2 -/- genotype.**

PCR primers:

FW: 5’- CAAATGCCGGCAGTCTGATG-3’

RV: 5’- GACAGCTGAGGGCAGTGAG-3’

Internal primers:

FW: 5’- GACAGCTGAGGGCAGTGAG-3’

RV: 5’- GACTCCAGAATTGGAGGCATGATG-3’

Sequencing primer:

FW: 5’- CACAAGATAAAAACGGTCTGTAACACTG-3’
