# Supplementary material for: Glycosylated clusterin species facilitate Aβ toxicity in human neurons
Source: Sci Rep. 2022 Nov 3;12:18639. doi: 10.1038/s41598-022-23167-z (PMC9633591; doi:10.1038/s41598-022-23167-z)
Supplement: Supplementary file 10 — Supplementary Table 3. [file 41598_2022_23167_MOESM10_ESM.docx]

**Supplementary table 3: List of immunocytochemistry antibodies used in this study.**

| **Primary antibody** | **Species, dilution** | **Company** | **Secondary antibody** |
| --- | --- | --- | --- |
| MAP2 | Chicken, 1:1000 | Abcam, ab5392 | Goat anti chicken AlexaFluor 647, Life Technologies, A-21449 |
| CTIP2 | Rat, 1:500 | Abcam, ab18465 | Donkey anti rat AlexaFluor 488, Life Technologies, A21208 |
